# Cardiac Abnormalities in Patients With Severe Fever With Thrombocytopenia Syndrome: A Systematic Review

**DOI:** 10.1093/ofid/ofad509

**Published:** 2023-10-13

**Authors:** Qiaoling Liu, Mingming Yang, Shichun Shen, Chen Gong, Zuyong Lan

**Affiliations:** School of Cardiovascular and Metabolic Health, University of Glasgow, Glasgow, UK; British Heart Foundation Glasgow Cardiovascular Research Centre, University of Glasgow, Glasgow, UK; Department of Cardiology, Zhongda Hospital, School of Medicine, Southeast University, Nanjing, China; Department of Cardiology, The First Affiliated Hospital of University of Science and Technology of China, Hefei, China; Department of Paediatrics, The First Affiliated Hospital of Anhui Medical University, Hefei, China; Wolfson Wohl Translational Cancer Research Centre, Institute of Cancer Sciences, University of Glasgow, Glasgow, UK

**Keywords:** cardiac abnormality, human, severe fever with thrombocytopenia syndrome

## Abstract

Since the identification of severe fever with thrombocytopenia syndrome virus (SFTSV) in 2010, there has been an increase in reported cases in China and other Asian countries. Cardiac abnormalities are highly prevalent in SFTS patients. We searched 5 Chinese and international databases for published SFTS articles and extracted patient characteristics, cardiac complications, electrocardiography findings, and imaging findings. Twenty-seven studies were identified, covering 1938 patients and 621 cardiac abnormalities. Arrhythmia was the most prevalent, reported in 24 studies and 525 cases, with a prevalence of 27.09%. The 2 major types of arrhythmias were bradycardia and atrial fibrillation. Heart failure was the second most prevalent abnormality, with 77 cases. Changes in the ST segment and T wave were the most common. Valve regurgitation, reduced ejection fraction, and pericardial effusion were also documented. We recommend that physicians pay close attention to newly onset arrhythmia and structural heart disease in SFTS patients.

Severe fever with thrombocytopenia syndrome (SFTS) has emerged as a noteworthy infectious disease in Asian countries in recent years. SFTS cases were first noticed in China in 2007, with symptoms resembling human granulocytic anaplasmosis (HGA) [1]. Followed by heightened surveillance, the isolation and identification of a new phlebovirus in the Bunyaviridae family was completed in 2010 [2, 3]. In 2020, the new virus was officially named *Dabie bandavirus* (renamed *Bandavirus dabieense* in 2011), with synonyms including *Huaiyangshan banyangvirus* and severe fever with thrombocytopenia syndrome virus (SFTSV). Currently, SFTSV has at least 6 known genotypes (A to F), and the distribution of these genotypes varies among different regions/countries [4].

The main transmission of SFTS is through bites from the Asian longhorned tick (*Haemaphysalis longicornis*) [5]. However, there have also been reports of human infection through contact with respiratory secretions or blood from patients, resulting in localized outbreaks [1]. In addition to its endemicity in the central and southern provinces of China, such as Henan, Jiangsu, and Zhejiang, there have been recent reports of its occurrence in Japan and South Korea.

The virus's pathogenesis entails a cytokine storm leading to inflammation, coagulation, and potential multi-organ failure, featuring serum biomarkers of the liver, kidney, and heart [6, 7]. The elevation of heart biomarkers (eg, C-reactive protein) hints at cardiac impairment and possible abnormalities.

The clinical presentation of SFTS lacks specific characteristics. The predominant symptoms comprise fever, anorexia, fatigue, and muscle soreness. As the disease progresses, most patients experience cardiac, hepatic, and renal dysfunction, often necessitating admission to the intensive care unit (ICU). Severe cases can have central nervous system syndrome, multi-organ failure, and disseminated intravascular coagulation, with mortality rates ranging from 7% to 30% [8].

Nevertheless, there are inconsistencies in the current literature regarding cardiac abnormalities in SFTS patients. Li et al. examined the clinical features of 2096 SFTS patients hospitalized in a major medical facility in Henan Province between 2011 and 2017. No cardiac abnormalities were reported, though the creatine kinase (CK) levels tested at admission in deceased patients were elevated compared with those who survived [9]. In another retrospective review of 115 hospitalized patients with SFTSV infection, 106 patients underwent electrocardiogram tests, and 23.6% of patients (25 people) showed arrhythmia. Of 31 patients who were tested for troponin, 58.1% (18 people) had elevated levels. Upon admission, abnormal elevation of cardiac biomarkers such as CK, creatine kinase-MB (CK-MB), and lactate dehydrogenase (LDH) could be detected in >70% of patients, reflecting a high prevalence of myocardial damage [10].

A number of case reports have also documented the occurrence of cardiac complications, including atrial fibrillation (a-fib), sinus bradycardia, and others, after the onset of symptoms [11–13]. The current expert consensus does not provide a detailed analysis of cardiac involvement in SFTS patients [2]. Recognizing and preventing early-onset cardiac abnormalities in SFTS patients is crucial due to the substantial threat posed to their lives. The rising number of reported SFTS cases in China and the Asian region further emphasizes the importance of investigating cardiac complications in SFTS patients.

In the above context, this review aims to provide a thorough analysis of the cardiac abnormalities observed in patients with SFTS. By consolidating the available evidence, this review seeks to offer general insights into the cardiac impacts of SFTS, hoping to improve patient care and advance our understanding of this challenging infectious disease.

## METHODS

This review has been registered on PROSPERO (CRD42023434520). We adhered to the Preferred Reporting Items for Systematic Reviews and Meta-Analysis Statement (PRISMA) [14] for this review.

One author (Q.L.) conducted thorough literature searches across multiple databases, including China National Knowledge Infrastructure (CNKI), Wanfangdata, Embase, Medline, and PubMed. The searches encompassed manuscripts published from January 1, 2010, to June 16, 2023. Case series and observational studies with longitudinal and cross-sectional designs were included. To ensure our findings’ reliability and accuracy, we excluded the following studies: (1) animal and in vitro studies; (2) studies categorized as case reports, comments, conference papers, editorials, meta-analyses, notes, preprints, or reviews; (3) studies in which SFTS diagnosis approaches were unclear; (4) studies that did not report cardiac complications. A search for gray literature was not performed due to resource constraints and challenges accessing it. Furthermore, the variable quality and lack of peer review in gray literature raised concerns about the validity and consistency of the data. The detailed search terms can be found in Supplementary Table 1. Two authors (Q.L. and M.Y.) independently evaluated the eligibility of the identified studies for inclusion in this review. In cases of disagreement, discussions were held with 2 additional authors (S.S. and C.G.) to reach a consensus.

### Identification of Cardiac Abnormalities

In this review, we relied on the diagnosis text recorded in each included study to identify cardiac abnormalities. General definitions of 4 major cardiac abnormalities are listed below for quick reference: (1) bradycardia: a heart rate <60 beats/min; (2) tachycardia: a heart rate ≥100 beats/min; (3) heart failure: a clinical syndrome due to structural and/or functional abnormality of the heart, characterized by a range of typical clinical symptoms (eg, breathlessness and fatigue) potentially accompanied by signs (eg, elevated jugular venous pressure, pulmonary crackles, and peripheral edema); (4) myocarditis: a sudden inflammatory injury of the myocardium mainly due to infectious factors (primarily viruses and bacteria) and/or noninfectious factors (eg, autoimmune disorders and allergens).

### Quality Assessment

Two authors independently evaluated the quality of the included studies. As all the studies covered were case series, we adopted and visualized Joanna Briggs Institute's Critical Appraisal Checklist for Case Series [15]. In cases where there were differences in judgment between the 2 reviewers, discrepancies were resolved through discussions with a third author, who provided assistance as required.

### Data Synthesis

A predesigned table was used to extract information on the first author's family name, publication year, country of study, size of the study, patient age, patient sex, cardiac complications, electrocardiography (ECG) findings, imaging findings, and mortality rate. If only stratified data (eg, by sex) were available in a manuscript, then all the stratified data would be extracted. For studies that covered the same population, only the study with the largest study size was selected to avoid duplicate records. We applied established methods to convert the median (interquartile range [IQR]) of the reported data to the mean (standard deviation [SD]) [16].

## RESULTS

### Identification of Studies

**Figure 1.**
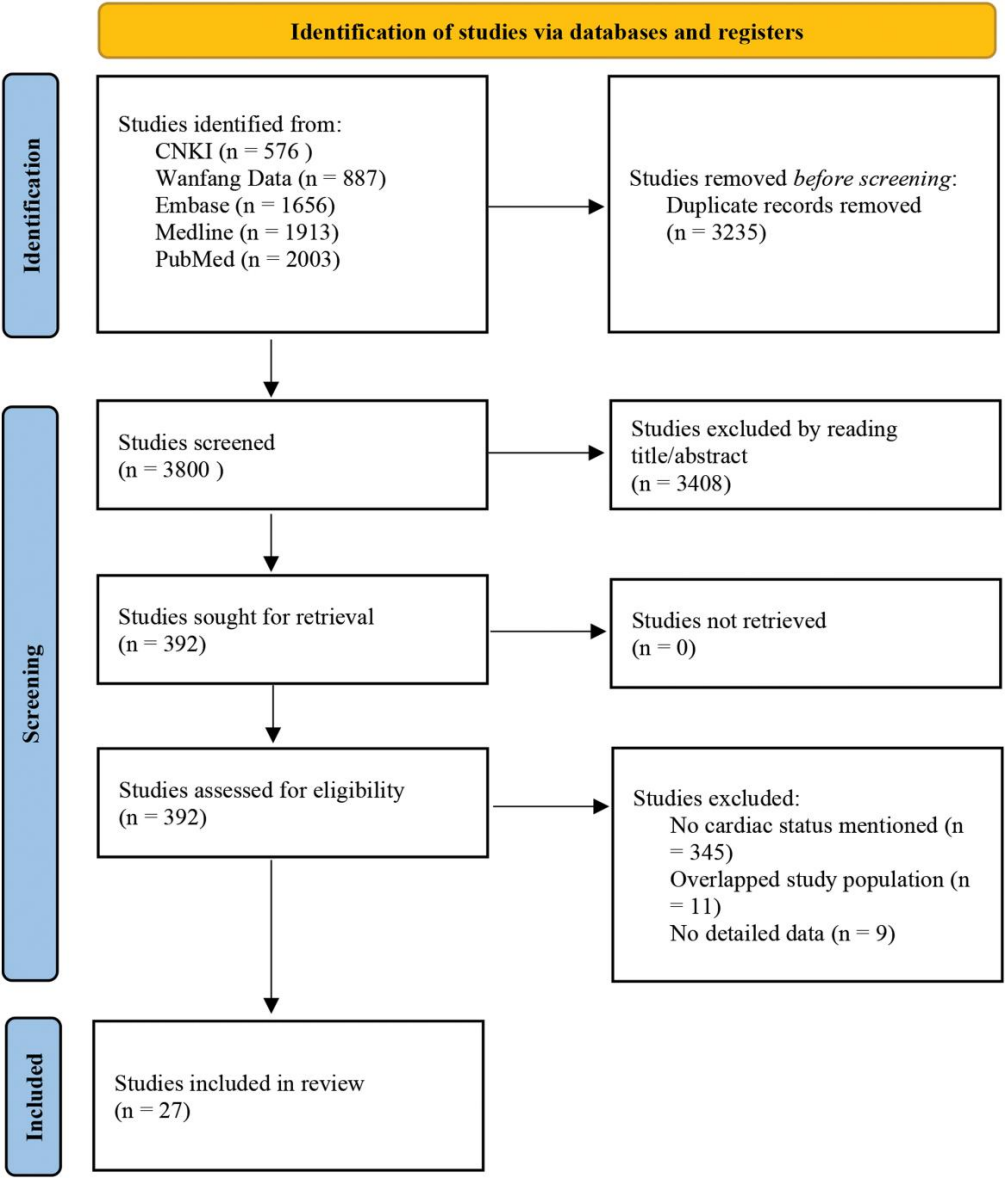
Preferred Reporting Items for Systematic Reviews and Meta-Analysis flow of study selection.

After removing duplicate studies, a total of 3800 potentially relevant studies were identified. From the initial screening of titles and abstracts, 392 studies were selected for full-text screening. After thoroughly examining full texts, a final selection of 27 studies was included in this review [10, 12, 13, 17–40]. All 27 studies were case series (Figure 1).

### Characteristics of the Included Studies

The 27 identified studies encompassed a population of 1938 SFTS patients, of whom 54.33% were male (1053 people). Individual study sample sizes ranged from 6 to 216, with a median of 65 and an interquartile range of 85. All the patients had the diagnosis of SFTSV infection confirmed by either positive reverse transcriptase polymerase chain reaction (RT-PCR) or positive antibody serology test results.

The mean (SD) age of reported patient groups was 60.86 (5.95) years. Twenty-four out of 27 studies concentrated on Chinese patients; 91.7% (22 out of 24) of studies that focused on Chinese patients were from 4 interconnected provinces: Shandong, Jiangsu, Zhejiang, and Anhui. Three manuscripts examined South Korean patients [23, 27, 36].

One study exclusively focused on deceased patients [24], another was on patients admitted to the ICU [35], and the remaining studies covered general patients.

In general, the current research lacks comprehensive documentation of cardiac abnormalities. Much research used only a few sentences to describe the cardiac condition of patients. Therefore, the extraction of information was very limited. A total of 621 cardiac abnormalities in the 1938 SFTS patients were reported, with a prevalence of 32.04%. The most common abnormality was arrhythmia, occurring at a prevalence of 27.09%. Heart failure was the second most common complication, which accounted for 3.97% of all patients. Additionally, 19 cases of various complications, such as myocarditis, pericardial effusion, acute coronary syndrome (ACS), and angina, were reported. Seven studies involving 442 patients reported 194 ECG changes, primarily manifesting as ST segment/T wave changes. Furthermore, 1 study reported imaging findings with regurgitations, and another study reported pericardial effusion (Table 1).

**Table 1.** Characteristics of the Studies Included in the Review (n = 27)

Author	Year	Country	Patient Size	Patient Age, y	Male, %	Cardiac Complication (No. of Patients)	ECG Changes in Detail (No. of Patients)	Imaging Findings (No. of Patients)	Mortality Rate, %
Sun et al. [17]	2012	China	12	Mean (SD): 55 (3)	58.33	Bradycardia (9)	NA	NA	NA
Wei et al. [18]	2012	China	6	Mean (SD): 52 (14)	50	PAC (1)	NA	Aortic regurgitation (1)	16.67
						Sinus bradycardia (1)		Mitral regurgitation (1)	
								Tricuspid regurgitation (1)	
Deng et al. [10]	2013	China	115	Mean (range): 55 (17–89)	65.20	Acute LVF (5)	T wave flat/diphasic/inverted without ST segment changes (8)	Pericardial effusion (5)	12.20
						A-fib (6)			
						AV block (2)			
						PVC (5)			
						Sinus bradycardia (5)			
						SV tachycardia (8)			
						V-fib (1)			
Han et al. [19]	2015	China	22	Median (range): 59 (41–75)	50	A-fib (2)	NA	NA	NA
						Sinus bradycardia (9)			
Ma et al. [12]	2015	China	65	Mean (range): 54.3 (23–72)	63.10	A-fib (2)	T wave changes (5)	NA	15.40
						PAC (3)	ST-T changes (14)		
						Sinus bradycardia (21)			
						Sinus tachycardia (4)			
Qin et al. [20]	2015	China	12	Range: 31–79	75	A-fib (1)	NA	NA	16.67
						Atrial tachycardia (1)			
						PAC (1)			
						PVC (1)			
						Sinus bradycardia (1)			
Shao et al. [21]	2015	China	107	Mean (SD): 59.19 (11.35)	63.55	A-fib (9)	ST segment depression/diphasic/inverted with T wave changes (28)	NA	NA
						Atrial flutter (1)			
						PAC (1)			
						PVC (1)	Prolonged QT interval (3)		
						Sinus arrhythmia (2)			
						Sinus bradycardia (11)			
						Sinus tachycardia (3)			
						SV tachycardia (2)			
Zhang and Gao [22]	2015	China	25	Mean (SD): 50.5 (11)	60	Angina (1)	NA	NA	0
						Myocarditis (6)			
Zhou et al. [13]	2015	China	68	Mean (SD): 58 (13.2)	47.06	A-fib (10)	NA	NA	19.12
						Atrial flutter (1)			
						Bradycardia (10)			
Choi et al. [23]	2016	Korea	120	Mean (SD): 68.33 (13.33)	50.80	Arrhythmia (14)	NA	NA	38.33
						Myocarditis (5)			
Ni et al. [24]^a^	2016	China	17	Mean (range): 61 (44–78)	76.50	A-fib (2)	ST-T changes (15)	NA	100
						Bradycardia (15)			
						PAC (2)			
						PVC (6)			
Shi et al. [25]	2016	China	6	Mean (range): 52 (28–66)	83	AV block (1)	ST segment changes (2)	NA	16.67
Chen et al. [26]	2017	China	42	Mean (SD): 58.9 (2.5)	61.90	A-fib (2)	ST-T changes (8)	NA	4.76
						Sinus tachycardia (17)	T wave changes (4)		
Oh et al. [27]	2017	Korea	53	Nonplasma exchange group, mean (SD): 67.4 (11.6)	Nonplasma exchange group: 55.20	ACS (2)	NA	NA	32.08
				Plasma exchange group, mean (SD): 62.5 (11.6)	Plasma exchange group: 62.50				
Cong [28]	2018	China	90	Mean (range): 67.27 (24–83)	68.89	A-fib (7)	ST-T changes (18)	NA	20
						PAC (1)	T wave changes (6)		
						PVC (1)			
						Sinus bradycardia (27)			
						Sinus tachycardia (1)			
Hu et al. [29]	2018	China	25	Mean (SD): 57.8 (12.66)	60	Arrhythmia (5)	NA	NA	20
Liu et al. [30]	2018	China	56	Mean (SD): 64 (9)	44.64	Arrhythmia (17)	NA	NA	28.57
Xia et al. [31]	2018	China	86	Mean (SD): 62 (10.82)	46.51	Bradycardia (6)	NA	NA	13.95
						Tachycardia (62)			
Chu et al. [32]	2019	China	40	Mean (SD): 65.7 (11)	47.50	A-fib (10)	NA	NA	17.50
						HF (7)			
Gao and Song [33]	2019	China	14	Mean (range): 64 (29–81)	57.14	Arrhythmia (4)	NA	NA	7.14
Shen [34]	2019	China	96	Mean (SD): 53.44 (7.74)	58.33	A-fib (8)	NA	NA	25
						Bradycardia (5)			
Nie et al. [35]^b^	2020	China	116	Mean (SD): 63 (9.1)	51.30	Arrhythmia (42)	NA	NA	43.10
						HF (44)			
Jung et al. [36]	2021	Korea	142	Mean (SD): 68.27 (10.59)	50.70	Arrhythmia (8)	NA	NA	23.24
Song et al. [37]	2022	China	216	Mean (SD): 66.54 (10.64)	54.17	Arrhythmia (41)	NA	NA	25
						HF (10)			
Wang et al. [38]	2022	China	86	Mean (SD): 60 (13.5)	46.50	Arrhythmia (22)	NA	NA	12.80
						HF (11)			
Wang et al. [39]	2022	China	198	With central neurological complication, mean (SD): 66.4 (8.6)	51.51	A-fib (25)	NA	NA	14.65
				Without central neurological complication, mean (SD): 58.4 (12.7)		PAC/PVC (15)			
Zhang and Zhang [40]	2022	China	103	With A-fib, mean (SD): 74.5 (7.8)	With A-fib: 40.70	A-fib (27)	NA	NA	NA
				Without A-fib, mean (SD): 66.5 (10.9)	Without A-fib: 39.40				

Abbreviations: ACS, acute coronary syndrome; A-fib, atrial fibrillation; AV block, atrioventricular block; ECG, electrocardiography; HF, heart failure; ICU, intensive care unit; NA, not available; PAC, premature atrial complex; PVC, premature ventricular complex; V-fib, ventricular fibrillation.

^a^Study on deceased patients only.

^b^Study on ICU-admitted patients only.

### Quality Assessment of Included Studies

**Figure 2.**
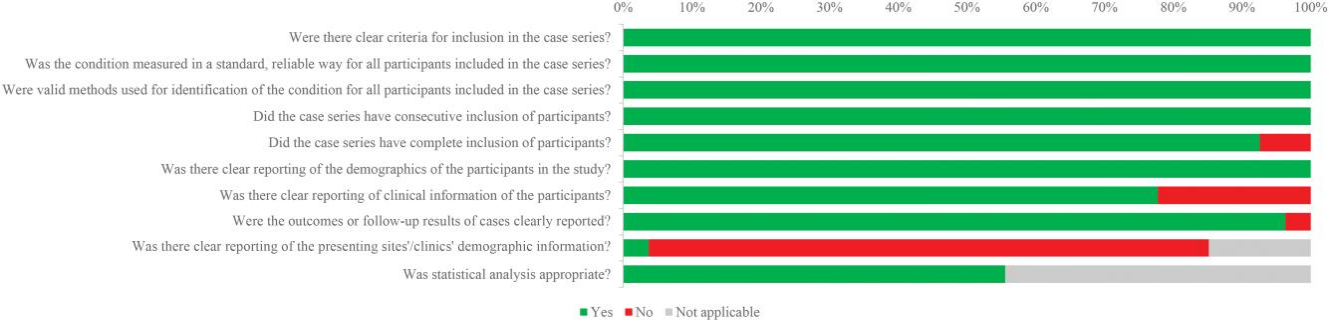
Summary of the quality assessment for selected studies using the JBI critical appraisal checklist for case series.

The majority of studies showed satisfactory quality. Six studies reported unclear lab results [17, 20, 22, 31, 33, 34], and 1 did not report patient outcomes [17]. Most studies did not report the demographic information of the presenting sites/clinics (Figure 2).

### Arrhythmia in SFTS Patients

Arrhythmia was the most common cardiac abnormality in the included studies. Twenty-four out of 27 studies reported arrhythmia patients, with 525 cases. Of all the cases, 155 (29.52%) have unspecified types of arrhythmia. For all the other arrhythmia cases, bradycardia (sinus/junctional) has the highest percentage at 22.86%, followed by 21.13% atrial fibrillation, 18.67% tachycardia (sinus/supraventricular), and 7.24% premature atrial/ventricular complex (PAC/PVC). Two cases of atrial flutter and 1 of ventricular fibrillation (v-fib) were also recorded. In a small study of 25 patients, Hu et al. [29] reported a positive association between arrhythmia and mortality [odds ratio [OR], 13.5; 95% CI, 1.340–135.983; *P* = .027). Still, this association was not observed after adjusting for multiple confounders. The association was also not observed in a large group covering 216 patients (OR, 3.29; 95% CI, 0.60–17.96; *P* = .168) [37]. In ICU-admitted patients, Nie et al. [35] showed no significant differences in the prevalence of arrhythmia between survivors and nonsurvivors (*P* = .665), although arrhythmia was slightly more common in nonsurvivors (38.8% vs 34.8%). Additionally, arrhythmia was positively associated with 30-day mortality (adjusted hazard ratio, 4.61; 95% CI, 1.42–14.94; *P* = .011) [36] and central neurological complications (OR, 21.598; 95% CI, 4.896–95.270; *P* < .001) (Table 2) [39].

**Table 2.** Summary of Reported Cardiac Complications in SFTS Patients

Category	Type	No. of Cases
Arrhythmia	Unspecified	155
	Bradycardia	120
	A-fib	111
	Tachycardia	98
	PAC/PVC	38
	Atrial flutter	2
	V-fib	1
Heart failure	Unspecified	72
	Acute LVF	5
Others	Myocarditis	11
	Pericardial effusion	5
	ACS	2
	Angina	1

Abbreviations: ACS, acute coronary syndrome; A-fib, atrial fibrillation; LVF, left ventricular failure; PAC, premature atrial complex; PVC, premature ventricular complex; SFTS, severe fever with thrombocytopenia syndrome; V-fib, ventricular fibrillation.

### Heart Failure in SFTS Patients

Seventy-seven heart failure cases were reported in 5 of 27 studies [10, 32, 35, 37, 38]. The distribution of heart failure among surviving and deceased patients was inconsistent, with 2 reports showing a larger share of cases in deceased patients [32, 37] and 1 study showing a higher prevalence in survivors [31]. No association between heart failure and mortality was found in the reports (Table 2).

### Other Complications in SFTS Patients

Eleven cases of myocarditis were reported in 2 studies [22, 23]; Choi et al. [23] reported no differences in the proportion of myocarditis between fatal and nonfatal patients. In addition, 5 cases of mild pericardial effusion were diagnosed through echocardiography [10]. Angina [22] and acute corona syndrome [27] were also observed, with 1 case each (Table 2).

### ECG Abnormalities in SFTS Patients

Seven studies reported 194 ECG abnormalities in 442 patients [10, 12, 21, 24–26, 28]. More than half of the reported changes were T wave changes, with a prevalence of 54.64% (106 out of 194). The T wave change could be flat, diphasic, inverted, or with/without ST segment changes. Changes in the ST segment were also commonly found (85 out of 194); the change could be depression, diphasic, or inverted. A prolonged QT interval was also reported (Table 2) [21].

## DISCUSSION

Our review delves into the cardiac abnormalities observed in patients with SFTS. From a pool of 1938 SFTS patients analyzed across 27 studies, 32.04% were found to have cardiac abnormalities. The most common cardiac abnormality was arrhythmia, noted in 27.09% of patients, followed by heart failure in 3.97%. Other cardiac complications, including myocarditis, pericardial effusion, acute coronary syndrome, and angina, were sporadically reported. ECG changes, primarily manifesting as alterations in the ST segment or T wave, were observed in several patients.

Notably, Cong [28] described that patients with atrial fibrillation can experience heart rates as high as 186 beats/min, while bradycardia patients can have rates as low as 39 beats/min. Particularly noteworthy is the report by Liu et al. [30], stating that among 56 patients, 17 developed arrhythmias 7 to 10 days after the onset of symptoms, despite showing no abnormalities in their initial electrocardiograms upon admission. This temporal sequence implies that SFTSV infection may be the underlying cause of the arrhythmia. The autopsy indicated the presence of SFTSV within cardiac cells, with high SFTSV-RNA copies (1.13 × 10^3^ copies/cell) [41]. Furthermore, B-cell-lineage lymphocytes infected with SFTSV have been widely detected in the heart [42]. Direct viral infection of cardiac cells likely played a significant role in inducing arrhythmia. An in vitro study revealed that SFTSV could target and infect endothelial cells, leading to increased permeability due to the disruption of intercellular junctions and inflammatory changes [43]. It is plausible that SFTSV infection interfered with ion channels in cardiac cells or led to immune-mediated damage to myocardial cells [44], resulting in the instability of cardiac electrical activity and subsequent development of arrhythmias. Some SFTS patients, despite not having SFTSV detected in their cardiac tissues, still exhibited symptoms such as heart failure or ventricular tachycardia. This phenomenon might be attributed to the hypercytokinemia induced by the viral infection [6], subsequently sparking a systemic inflammatory response [45, 46].

It is possible that the onset of cardiac abnormalities in SFTS patients might be due to comorbidities. In the studies included, hypertension and type 2 diabetes mellitus were common. These diseases are known risk factors for cardiovascular diseases [47, 48]. However, cardiac abnormalities were not studied as outcomes in all but 2 studies. Zhang et al. found that NT-proBNP and CK peak value were risk factors for newly onset atrial fibrillation [40], similar to Shao et al., who found that LDH and CK-MB were elevated in SFTS patients with abnormal ECGs [21]. Although no comorbidity data were presented or adjusted for, their study populations excluded patients with atrial fibrillation history, heart valve diseases, or diseases that can lead to secondary heart diseases (eg, hyperthyroidism). Several studies found no associations between comorbidities and SFTS severity/mortality [10, 23, 35]. Given the above research findings, it may be tenuous to conclude that cardiac abnormalities in SFTS patients are rooted in SFTS itself. Further studies using cardiac abnormalities as the study outcome are needed.

The manuscripts included in this review have observed the incidence of heart failure in SFTS patients. It is possible that a portion of heart failure cases may be attributed to persistent cardiac arrhythmias. Wei et al. [18] reported on mitral and tricuspid regurgitation among SFTS patients without a confirmed history of cardiovascular diseases, and both regurgitation and arrhythmias are recognized as risk factors for the development of heart failure. Additionally, several case reports have noted diffuse left ventricular wall motion depression and reduced ejection fraction (as low as 15%) detected through echocardiography [49, 50]. A decline in ejection fraction can also contribute to the onset of heart failure.

In nearly all SFTS patients, a significant increase in CK, CK-MB, and LDH levels, along with elevated troponin, upon admission has been documented [10, 24]. These elevated biomarkers reflected myocardial cell damage, with the degree of elevation increasing as the disease worsens. Myocardial injury may lead to myocarditis and pericardial effusion. One study on cytokine and chemokine in SFTS patients showed elevation of tumor necrosis factor (TNF)–α, interleukin (IL)-6, and RANTES. TNF-α is positively associated with disease severity [6].

TNF-α is a primary proinflammatory protein, with IL-6 and IFN-γ also playing crucial roles in triggering acute inflammatory responses. Their elevation could imply severe inflammatory responses inside human bodies, leading to endothelial dysfunction and cardiac abnormalities [51]. The abovementioned cases of angina and ACS were difficult to evaluate as there were only 3 patients and there was a lack of detailed clinical descriptions. However, myocardial injury likely also played a significant role in those cases. More detailed exploration is needed in future studies.

The current literature lacks detailed reports on ECG findings. Among the reported 194 ECG changes, 191 cases mentioned changes in the ST segment, T wave, or both, reflecting cardiac repolarization abnormalities. Some reports mentioned ST segment depression [10] and T wave flattening [21], which are often associated with myocardial ischemia and impairment. In addition to the findings, as mentioned earlier, ECG changes in SFTS patients may include T wave flattening in V_5,6_ [50] and ST elevation in II, III, aVf, and V_2-6_ [49, 50]. To the best of our knowledge, only 1 article has analyzed the differences between SFTS patients with and without ECG changes [21]. Although the article stated that SFTS patients with ECG abnormalities had higher levels of CK, CK-MB, and LDH and lower levels of serum sodium and calcium, the authors did not analyze ECG abnormalities as a research outcome after adjusting for potential confounding factors. Therefore, the conclusions of the article may not be robust. However, physiologically, the degree of myocardial impairment is directly related to the presence of ECG abnormalities. We believe that this is a gap in current SFTS research that requires well-designed studies to bridge.

The significant occurrence of cardiac abnormalities in SFTS patients, such as arrhythmia and heart failure, underscores the need to monitor these patients closely. Despite their high prevalence, cardiac abnormalities have rarely been treated as the study outcome in existing studies, indicating a gap that future research must address (eg, risk factors). More dedicated studies, perhaps with larger sample sizes, well-recorded medical records, and focused examination of each cardiac abnormality, could provide a clearer understanding. In the clinical context, recognizing these abnormalities early and tailoring management strategies, such as regular ECG monitoring, may be crucial for patient outcomes. The genomic variations of SFTSV are linked to differences in mortality and vary among countries [52, 53]. Still, we do not have information on whether certain genotypes are more predisposed to causing cardiac complications than others. This lack of genotype-specific data further highlights an area ripe for exploration in future research endeavors.

The findings of this review should be considered in light of its limitations. First, the majority of the studies included in this research were based on Chinese patients, while SFTS has also been reported in other Asian countries such as Japan and Korea. The generalizability of our findings to Asian populations may be limited. Second, despite our extensive search in Chinese and international databases, we did not find relevant studies investigating cardiac abnormalities as the primary outcome, controlling for confounding factors. Consequently, we could not comprehensively analyze the demographic and clinical characteristics of patients with a higher risk of cardiac abnormalities. In the context where SFTS cases are less common, conducting collaborative multicenter studies would greatly contribute to our understanding of the topic. Third, the literature included in this review provided limited descriptions of cardiac abnormalities, which restricted our ability to delve deeper into the topic. We incorporated some case reports in the “Discussion” section to facilitate the analysis. However, it is important to acknowledge that case reports are susceptible to publication bias. We limited inclusion to studies that reported cardiac abnormalities, noting that there are studies that did not observe such abnormalities in SFTS patients. Therefore, the actual prevalence of cardiac abnormalities may be lower than our findings. Fourth, we did not include gray literature in our research. Adding gray literature may offer insights into emerging trends and novel findings that may not yet have made their way into mainstream publications. Last, the identification of cardiac abnormalities was based on reported diagnosis. It is possible that diagnostic criteria varied among included studies, with a mixture of clinical and pathological diagnoses. Readers should exercise caution when interpreting our conclusions.

Despite the abovementioned limitations, this systematic review may contribute to the understanding of cardiac abnormalities in SFTS patients. By thoroughly searching and analyzing available research from multiple databases, our review provided a general overview for physicians. While we acknowledge the presence of limited information and potential biases, we view our findings as a valuable starting point that may inform future studies.

In conclusion, our study revealed widespread cardiac abnormalities in SFTS patients, especially arrhythmia. Clinical care for this subset of patients warrants attention. Future research, meticulously designed with cardiac abnormalities as study outcomes, is needed to explore potential risk factors and pathogenesis. Additionally, the clinical manifestations between different genotypes of SFTSV also deserve attention.

## Supplementary Material

ofad509_Supplementary_Data

## Supplementary Data

Supplementary materials are available at *Open Forum Infectious Diseases* online. Consisting of data provided by the authors to benefit the reader, the posted materials are not copyedited and are the sole responsibility of the authors, so questions or comments should be addressed to the corresponding author.
