# Supplementary material for: Cardiac Abnormalities in Patients With Severe Fever With Thrombocytopenia Syndrome: A Systematic Review
Source: Open Forum Infect Dis. 2023 Oct 13;10(11):ofad509. doi: 10.1093/ofid/ofad509 (PMC10627340; doi:10.1093/ofid/ofad509)
Supplement: ofad509_Supplementary_Data [file ofad509_supplementary_data.docx]

**Supplementary Table 1. Search Terms**

|  | **PubMed** | **Number of articles** |
| --- | --- | --- |
| 1 | (Severe fever with thrombocytopenia syndrome[Title/Abstract]) OR (novel bunyavirus[Title/Abstract]) OR (bunya*[Title/Abstract]) OR (SFTS*[Title/Abstract]) | 4257 |
| 2 | ("2010/01/01"[Date - Publication] : "3000"[Date - Publication]) | 15776764 |
| 3 | #1 AND #2 | 2937 |
| 4 | (animals[MeSH Terms] NOT humans[MeSH Terms]) | 5124951 |
| 5 | #3 NOT #4 | 2345 |
| 6 | #5 NOT ("editorial"[pt] OR "case reports"[pt] OR "comment"[pt] OR "Meta-Analysis"[pt] OR "Review"[pt] OR "Preprint"[pt]) | 2003 |
|  |  |  |
|  | **Embase 1947-Present, updated daily** |  |
| 1 | (Severe fever with thrombocytopenia syndrome or novel bunyavirus or SFTS* or bunya*).ti,ab. | 4786 |
| 2 | limit 1 to yr="2010 -Current" | 3322 |
| 3 | 2 NOT ((exp animal/ or nonhuman/) NOT exp human/) | 2267 |
| 4 | 3 NOT (conference abstract or conference paper or conference review).pt. | 1876 |
| 5 | 4 NOT (editorial or comment or note or case report or review).pt. | 1656 |
|  |  | |
|  | **Ovid MEDLINE(R) ALL 1946 to June 16, 2023** | |
| 1 | (Severe fever with thrombocytopenia syndrome or novel bunyavirus or SFTS* or bunya*).ti,ab. | 4076 |
| 2 | limit 1 to yr="2010 -Current" | 2758 |
| 3 | 2 NOT ((exp animal/ or nonhuman/) NOT exp human/) | 2216 |
| 4 | 3 NOT (conference abstract or conference paper or conference review).pt. | 2216 |
| 5 | 4 NOT (editorial or comment or note or case report or review).pt. | 1913 |
|  |  |  |
|  | **China National Knowledge Infrastructure (CNKI)** |  |
|  | (主题=新型布尼亚病毒) OR (关键词=布尼亚病毒) OR (关键词=发热伴血小板减少综合征) | 576 |
|  |  |  |
|  | **Wanfangdata** |  |
|  | (主题:(新布尼亚病毒) or 题名或关键词:(新型布尼亚病毒) or 题名或关键词:(发热伴血小板减少综合征)) and Date:2010-* | 887 |
